# Enhancing Glucose Biosensing with Graphene Oxide and Ferrocene-Modified Linear Poly(ethylenimine)

**DOI:** 10.3390/bios14040161

**Published:** 2024-03-28

**Authors:** Jirawan Monkrathok, Pattanaphong Janphuang, Somphong Suphachiaraphan, Sastiya Kampaengsri, Anyanee Kamkaew, Kantapat Chansaenpak, Sireerat Lisnund, Vincent Blay, Piyanut Pinyou

**Affiliations:** 1 School of Chemistry, Institute of Science, Suranaree University of Technology, 111 University Ave., Nakhon Ratchasima 30000, Thailand; jirawan@g.sut.ac.th (J.M.); sastiya.ks@sut.ac.th (S.K.); anyanee@sut.ac.th (A.K.); 2 Synchrotron Light Research Institute (Public Organization), 111 University Ave., Nakhon Ratchasima 30000, Thailand; pattanaphong@slri.or.th (P.J.); somphong@slri.or.th (S.S.); 3 National Nanotechnology Center, National Science and Technology Development Agency, Thailand Science Park, Pathum Thani 12120, Thailand; kantapat.cha@nanotec.or.th; 4 Department of Applied Chemistry, Faculty of Science and Liberal Arts, Rajamangala University of Technology Isan, 744 Suranarai Rd., Nakhon Ratchasima 30000, Thailand; sireerat.in@rmuti.ac.th; 5 Department of Microbiology and Environmental Toxicology, University of California at Santa Cruz, Santa Cruz, CA 95064, USA

**Keywords:** glucose biosensor, glucose dehydrogenase, redox polymer, graphene oxide, flow injection

## Abstract

We designed and optimized a glucose biosensor system based on a screen-printed electrode modified with the NAD-GDH enzyme. To enhance the electroactive surface area and improve the electron transfer efficiency, we introduced graphene oxide (GO) and ferrocene-modified linear poly(ethylenimine) (LPEI-Fc) onto the biosensor surface. This strategic modification exploits the electrostatic interaction between graphene oxide, which possesses a negative charge, and LPEI-Fc, which is positively charged. This interaction results in increased catalytic current during glucose oxidation and helps improve the overall glucose detection sensitivity by amperometry. We integrated the developed glucose sensor into a flow injection (FI) system. This integration facilitates a swift and reproducible detection of glucose, and it also mitigates the risk of contamination during the analyses. The incorporation of an FI system improves the efficiency of the biosensor, ensuring precise and reliable results in a short time. The proposed sensor was operated at a constant applied potential of 0.35 V. After optimizing the system, a linear calibration curve was obtained for the concentration range of 1.0–40 mM (R^2^ = 0.986). The FI system was successfully applied to determine the glucose content of a commercial sports drink.

## 1. Introduction

Glucose biosensors are an essential tool in medical diagnostics and monitoring, playing a crucial role in managing and understanding conditions such as diabetes [1,2]. Electrochemical biosensors are particularly suitable for measuring physiological glucose levels as they can be miniaturized and integrated into wearable devices [3] and are compatible with different biofluids, such as blood, interstitial fluid, saliva, sweat, and urine [4,5].

A key component of electrochemical glucose biosensors is the biorecognition element, which selectively catalyzes the electrooxidation of glucose [6]. Oxidoreductase enzymes of different classes have been used, such as glucose oxidase (GOx), which has excellent substrate specificity and thermal stability [7,8,9] but requires molecular oxygen and leads to H_2_O_2_ formation and enzyme deactivation [10]. Another class is glucose dehydrogenases (GDHs). Instead of oxygen, they leverage a cofactor (PQQ, FAD, or NAD) [11]. While PQQ- and FAD-dependent GDHs suffer from broad substrate specificity [12], NAD-dependent GDHs possess a better selectivity towards glucose oxidation [11]. Unlike PQQ and FAD-GDHs, the NAD^+^ cofactor binds transiently to the protein [13,14].

The NADH molecule is rich in electrons and forms in stoichiometric relationship to the oxidized glucose. Harvesting electrons from NADH thus enables the quantification of glucose and the regeneration of NAD^+^. The redox reaction involving the NAD^+^ cofactor involves two electrons and one proton.

NAD^+^ + H^+^ + 2e^−^ ⇌ NADH

β-*D*-glucose + NAD^+^ ⇌ *D*-glucono-1,5-lactone + NADH + H^+^

The direct oxidation of NADH on the bare electrode surface requires the application of high overpotentials, which leads to undesired fouling [15,16]. One solution is to employ redox mediators, which enable the oxidation of NADH at lower overpotentials [17]. Redox mediators can be freely diffused or immobilized on the electrode surface [18]. There are multiple immobilization options, including electropolymerization [19,20], mediator-loaded electrospun nanofibers [21], and mediator-enriched carbon paste [22]. One approach is to covalently attach the mediator as pedant groups in a polymer backbone [23]. Several redox mediators have been bound to polymer backbones and co-entrapped with NAD-dependent redox enzymes, including phenazines [23,24] and metal complexes [13,25,26,27]. Among these, ferrocene has high chemical stability and low reorganization energy, which allows for a fast electron transfer through a self-exchange reaction [28,29].

Using ferrocene and its derivatives as freely diffusing redox mediators can result in unstable operation, as they do not stably adsorb on the electrode and their oxidized forms are very soluble [30,31]. Thus, they are frequently immobilized on the electrode surface instead [32,33]. Ferrocene has been immobilized on a glassy carbon electrode using Nafion, conferring improved stability to an electrode modified with glucose oxidase [31]. Ferrocene can be covalently bound to multiple polymer backbones, allowing flexibility in the design of biosensors [34]. Linear poly(ethylenimine) (LPEI) offers a positively charged polymer backbone to immobilize enzymes or nanomaterials with opposite charges [35] and amino groups for polymer crosslinking [36]. Several redox enzymes have been successfully employed with ferrocene-modified LPEI as a redox mediator [28,37,38].

Another strategy to improve sensor performance is to introduce nanomaterials [39,40,41,42] that increase the electroactive surface area and improve the electrical communication between the enzyme and the electrode surface [14,43]. Graphene oxide (GO) is often favored because of its biocompatibility and hydrophilicity [44,45], and its carboxylic groups can also interact with polycationic polymers [46]. Due to the abundance of oxygen-containing functional groups, GO is a negatively charged particle [47]. This enables the electrostatic interaction between GO and positively charged materials [48]. GO has been combined with a range of nanomaterials to improve the performance of electrochemical biosensors [49].

In this study, we designed and optimized a glucose biosensor utilizing a screen-printed electrode (SPE) modified with the GDH enzyme. To increase the electroactive surface area and the electron transfer efficiency, we introduced GO and ferrocene-modified linear poly(ethylenimine) (LPEI-Fc) on the biosensor surface. This design exploits the electrostatic interaction between both materials to obtain a stable enzyme–polymer film attached to the electrode. Moreover, GO also improves electrical conductivity and provides a large area for enzyme immobilization. The result is an overall improvement in glucose sensitivity. We also integrated the developed glucose sensor into a flow injection system. This integration can mitigate the risk of contamination and improve the precision and reliability of the measurements [50].

## 2. Materials and Methods

### 2.1. Reagents and Materials

Glucose dehydrogenase (GDH) from *Pseudomonas* sp. (≥200 U mg^−1^), nanocolloidal graphene oxide (2 mg mL^−1^), and Poly(2-ethyl-2-oxazoline) were purchased from Sigma Aldrich. *D*-glucose, β-nicotinamide adenine dinucleotide in oxidized form (NAD^+^, >95.0%), borane *tert*-butylamine complex, 3-Bromopropionyl chloride, dimethylferrocene, and ethylene glycol diglycidyl ether (EGDE) were obtained from TCI Chemicals (Tokyo, Japan). Ferrocene-modified linear poly(ethylenimine) (LPEI-Fc), a redox polymer, was prepared according to a previously established procedure [51]. The screen-printed electrodes used in this work were 4 mm diameter carbon working electrodes from Metrohm Dropsens (Oviedo, Spain) (commercial reference DRP-110).

### 2.2. Apparatus

Electrochemical measurements (cyclic voltammetry and amperometry) were performed using an EmStat3+ Blue potentiostat (Palmsens, Houten, The Netherlands). A three-electrode setup was used, comprising a carbon working electrode, a carbon auxiliary electrode, a silver (Ag) reference electrode, and the modified GDH/Fc-LPEI/GO on carbon SPE as the working electrode. Cyclic voltammetry of the modified electrodes was carried out at a scan rate of 10 mV s^−1^ and an electrolyte volume of 10 mL. The potentials reported in this work correspond to potentials vs. an Ag pseudo-reference electrode. Electrochemical impedance spectroscopy (EIS) of the modified electrodes was performed using a Palmsens4 potentiostat. EIS of the electrode with different modifications was performed in a redox couple solution containing 5 mM [Fe(CN)_6_]^3−/4−^ in 0.1 M KCl at an open-circuit potential of 0.140 V. The charge transfer resistance of the electrodes was evaluated at frequencies in the range of 100,000–0.05 Hz with an AC amplitude of 5 mV. The surface topology of the modified electrodes was investigated with a field-emission scanning electron microscope (Zeiss AURIGA FE-SEM/EDX, Carl Zeiss NTS GmbH, Oberkochen, Germany) at 1 kV acceleration voltage and 30 μm aperture. Fourier transform infrared (FT-IR) spectroscopy was performed in transmission mode and used to characterize the chemical functionalities on the GO surface using a Spectrum 100 FT-IR spectrometer (PerkinElmer, Waltham, MA, USA).

**Scheme 1:**
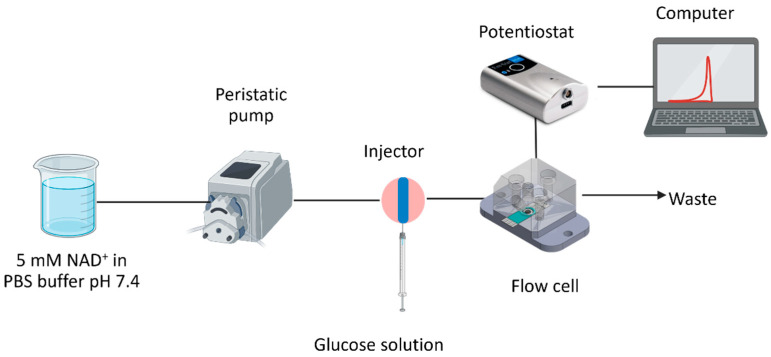
Flow injection system equipped with a GDH/LPEI-Fc/GO-modified electrode for the amperometric detection of glucose. The operating conditions include the following: applied potential = 0.35 V; carrier solution = 5 mM NAD^+^ in PBS buffer pH 7.4; and flow rate = 1.0 mL min^−1^.

The prepared glucose biosensor SPE was incorporated into a flow injection (FI) system, as depicted in Scheme 1. A peristaltic pump and a sample injector equipped with a 20 µL sample loop (Rheodyne, model 7725, IDEX Health & Science, Morrisville, NC, USA) were employed in the FI system. The modified SPE was placed in a custom-made electrochemical flow cell with a volume of 50 µL. The carrier solution used during the experiment was PBS buffer pH 7.4 containing 5 mM NAD^+^. The catalytic current response resulting from glucose oxidation was monitored amperometrically using an EmStat3+ Blue potentiostat at a constant applied potential of 0.35 V (vs. Ag). Signals were recorded as current vs. time profiles. Unless indicated otherwise, the peak height from individual injections was used to compare electrode performances. In all FI experiments, three injections were performed for each glucose solution measurement.

### 2.3. Preparation of the GDH/LPEI-Fc/GO-Modified Electrode

The GO suspension was diluted in DI water to attain a concentration of 1 mg mL^−1^. The GO suspension was sonicated for 90 min before being used for electrode modification. The GO/SPE was prepared by drop-casting 5 µL of the GO suspension on each SPE electrode. The electrodes were left to dry at room temperature prior to further modification.

**Scheme 2:**
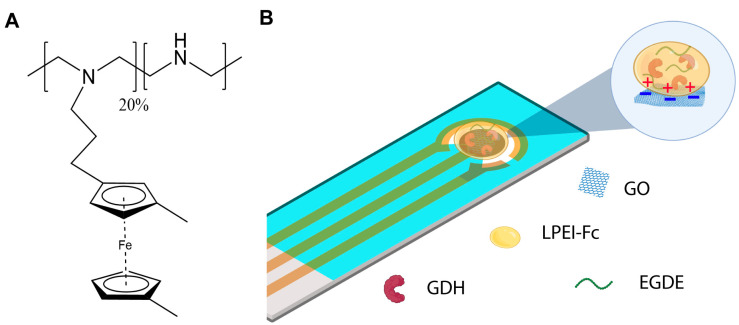
(**A**) Structure of the redox polymer ferrocene-modified linear poly(ethylenimine) (LPEI-Fc). (**B**) Components used for the GDH biosensor modification on a carbon screen-printed electrode. The modification leverages the electrostatic interaction between the negatively charged GO nanocolloid and the positively charged backbone of LPEI-Fc.

The solutions used to further modify the electrodes were prepared to have the following concentrations: GDH 10 mg mL^−1^, LPEI-Fc (12 mg mL^−1^), and EGDE (10% (*v*/*v*)) in a microcentrifuge tube (5 µg GDH, 30 µg LPEI-Fc, and 0.5 µg EGDE). The mixture of LPEI-Fc, GDH, and EGDE was thoroughly mixed using a vortex for 20 s and drop-casted on the GO/SPE or the SPE. The GDH/LPEI-Fc/GO electrodes thus prepared were allowed to dry at room temperature and stored overnight at 4 °C prior to measurement. Scheme 2 depicts the components used for the biosensor preparation in this work.

### 2.4. Measurement of Real Samples

The developed system was applied to the analysis of glucose contents in commercial sports drinks (SPONSOR, original, 250 mL, Prachin Buri, Thailand). The beverage samples were diluted by a factor of 20- to 30-fold with 5 mM NAD^+^ in PBS buffer pH 7.4 before measurement. The glucose concentration was determined by the standard addition method. The obtained values were compared with the results from a commercial glucometer (ACCU-CHEK Active, Roche Diabetes Care GmbH, Mannheim, Germany).

## 3. Results and Discussion

### 3.1. Electrochemical Characterization of the Electrodes with Different Modifications

**Figure 1:**
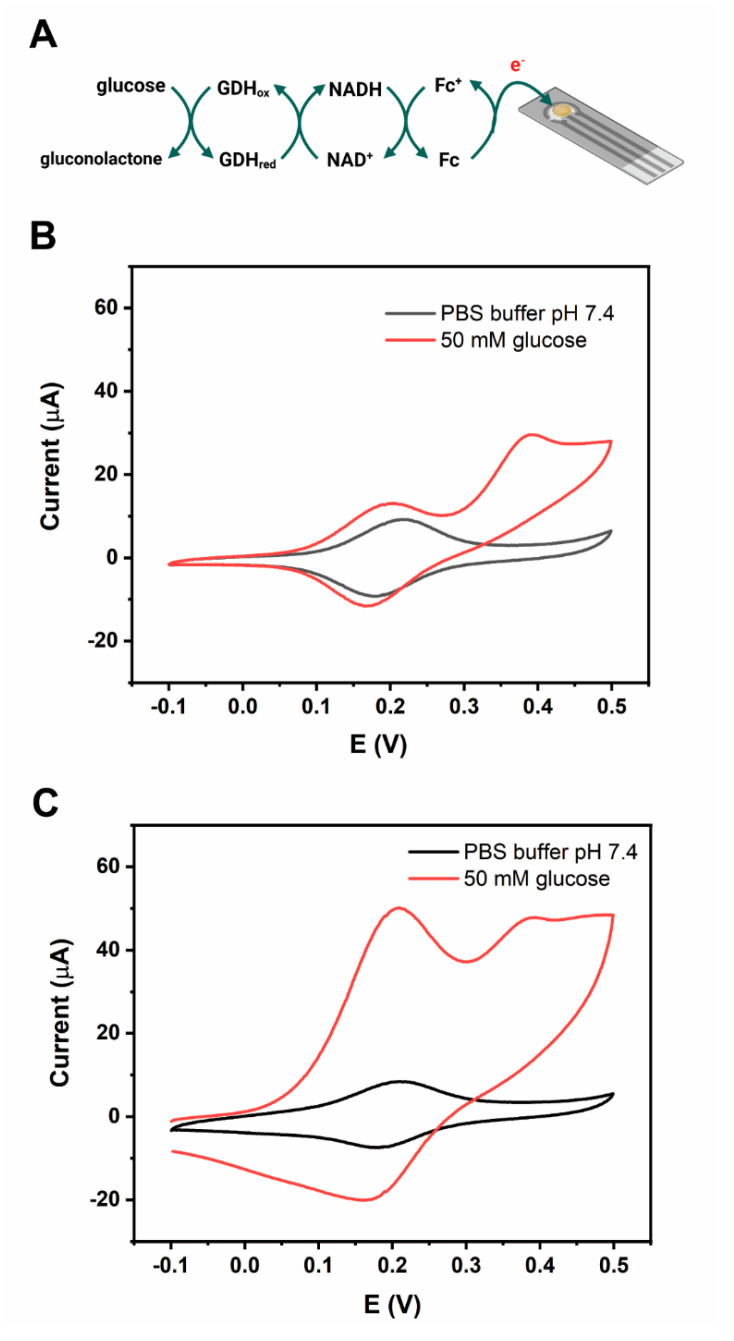
(**A**) Electron transfer pathway in the GDH glucose biosensor. The regeneration of NADH is facilitated by redox relays (ferrocene, Fc) in the LPEI-Fc polymer. Cyclic voltammograms of the GDH/LPEI-Fc/SPE (**B**) and the GDH/LPEI-Fc/GO/SPE (**C**) in the absence (black) and presence (red) of 50 mM glucose in 5 mM NAD^+^ in PBS buffer pH 7.4. Scan rate = 10 mV/s; air-equilibrated electrolyte.

Our NAD-GDH-modified electrode leverages a redox mediator to regenerate NADH from NAD^+^ during the measurement. Ferrocene (Fc) is incorporated into the side chain of the backbone of positively charged linear poly(ethylenimine) (LPEI). Fc serves as the redox mediator and facilitates the NADH oxidation process at a reasonably low potential. The electron transfer between the GDH enzyme and electrode surface is enhanced by a mediated electron transfer (MET) mechanism, through collisions of pendant ferrocene groups along the polymer backbone [52]. The electron transfer pathway of the modified electrode is depicted in Figure 1A.

In Figure 1B, the GDH/LPEI-Fc/SPE was investigated in the presence and absence of glucose in an electrolyte containing 5 mM NAD^+^. In the absence of glucose, the modified electrode showed a reversible redox potential of LPEI-Fc with an E_1/2_ of 0.20 V, excellent redox reversibility of ferrocene, and good agreement with previous reports [53,54]. Upon glucose addition, there were two oxidation peaks at 0.20 and 0.39 V. The first peak is attributed to the redox polymer, while the second one corresponds to the electrocatalytic oxidation of NADH facilitated by LPEI-Fc.

To improve the current response for NADH oxidation, we incorporated nanocolloidal graphene oxide (GO) onto the carbon surface of the SPE. This addition increases the specific surface area of the electrode, enabling the immobilization of a larger amount of GDH. Moreover, GO possesses oxygenated functional groups on its surface, such as epoxides, phenolic hydroxyl, carboxylic, and carbonyl groups. FT-IR measurements confirmed the availability of oxygen-containing functionalities on GO, as shown in Figure S1. The presence of these groups results in a hydrophilic surface favorable for the enzyme [44]. At neutral pH, GO displays a negatively charged surface [45], leading to a favorable electrostatic interaction between the GO surface and the positively charged LPEI redox polymer. The cyclic voltammograms (CVs) of the GDH/LPEI-Fc/GO/SPE are shown in Figure 1C. The oxidation redox peaks exhibited the same potential as those for the GDH/LPEI-Fc/SPE, but the catalytic currents significantly increased. This improvement is associated with the presence of nanocolloidal GO: its hydrophilicity and large surface area may improve enzyme dispersion and immobilization. Therefore, the SPE modified with GO was used for further experiments.

### 3.2. Surface Morphology of the Electrodes with Different Modifications

**Figure 2:**
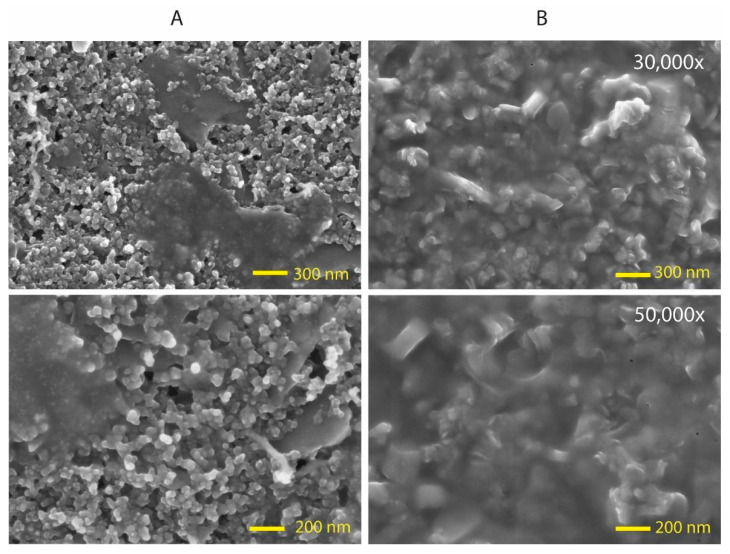
FE-SEM images of the SPCEs with different modifications at different magnifications: (**A**) GDH/LPEI-Fc/SPE and (**B**) GDH/LPEI-Fc/GO/SPE.

We investigated the effect of introducing GO on the surface of the carbon screen-printed electrode. Figure 2A shows that, in the absence of GO, the GDH–polymer film was partially adsorbed on the electrode surface. Notably, when GO was introduced, the GDH–polymer film adsorbed more uniformly and was more dispersed, as shown in Figure 2B. We interpret this as follows: in the absence of GO, the interaction between the film and the electrode surface is unfavorable. When GO is introduced, a more favorable electrostatic interaction between GO and the LPEI backbone is established. At the same time, GO can interact strongly with the carbon electrode surface by π-stacking interactions. The result is that the GDH–polymer film can strongly attach to the surface modified with GO.

An improved distribution of the GDH–polymer facilitates the stable interaction of the GDH enzyme with the polymer surface and the harvest of electrons during glucose oxidation. The results from this study agree well with the electrochemical characterization of the electrode.

### 3.3. Electrochemical Impedance Spectroscopy (EIS) Study

**Figure 3:**
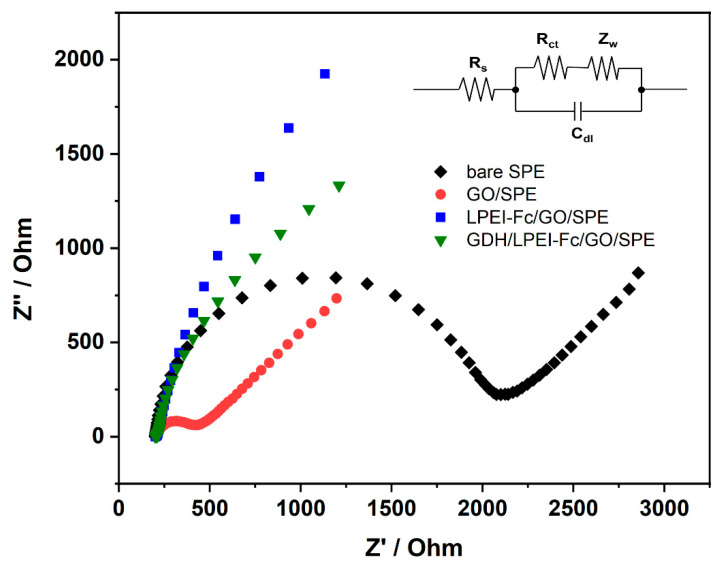
Nyquist plots of the SPE with different modifications and probed with a 5 mM redox couple of Fe(CN)_6_^3−/4−^ in 0.1 M KCl. The inset shows the equivalent Randles circuit used for modeling.

EIS is an electrochemical technique that allows for studying the impedance characteristics of electrical systems by applying a sinusoidal voltage perturbation whose frequency is varied over time [44]. The charge transfer resistance (R_ct_) of the electrodes with different modifications was evaluated by EIS. Measurements were conducted under a KCl electrolyte containing equimolar amounts of the redox couple probes hexacyanoferrate (III)/(II). The Nyquist plots obtained for each modification are shown in Figure 3.

To determine the R_ct_ values for each modification, we modeled our three-electrode system as a Randles circuit (inset). The results are shown in Table 1. The bare carbon SPE showed the largest R_ct_ value. After the incorporation of GO on the SPE surface, the R_ct_ value drastically decreased thanks to the improved electrical conductivity and increased specific surface area conferred by GO [42]. Upon introduction of the LPEI-Fc redox polymer on the GO/SPE, the R_ct_ value decreased further, evidencing an improved electrical conductivity compared to the GO/SPE setup. It is possible that the positively charged backbone of LPEI-Fc interacts favorably with the negatively charged redox couple and facilitates its diffusion to the electrode surface. By contrast, when the GDH enzyme was modified on the LPEI-Fc/GO/SPE, the R_ct_ value slightly increased, which is attributed to the non-conductive protein component increasing the interfacial resistance. The EIS results show that both GO and LPEI-Fc had a positive effect on improving electrical conductivity.

### 3.4. Optimization of the Modified Electrodes

**Figure 4:**
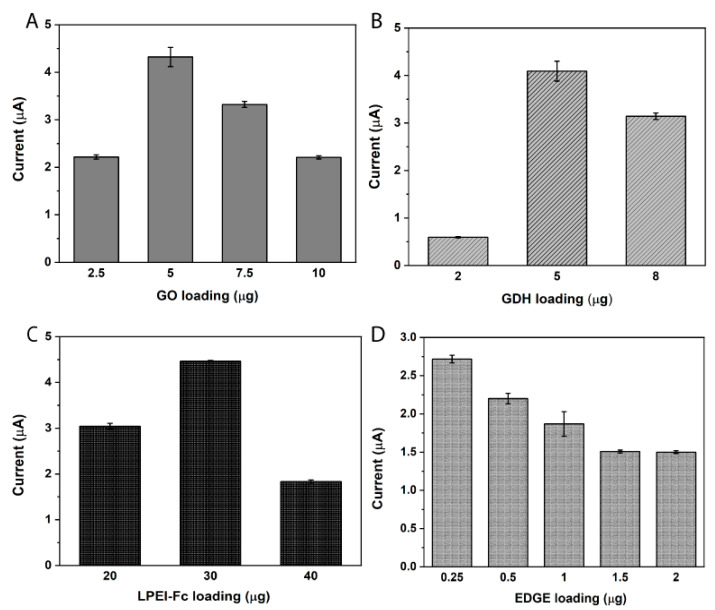
Effect of different component loadings on the current response to 40 mM glucose measured with the GDH/LPEI-Fc/GO/SPE: (**A**) GO (5 µg GDH, 30 µg LPEI, 0.25 µg EGDE), (**B**) GDH (5 µg GO, 30 µg LPEI, 0.25 µg EGDE), (**C**) LPEI-Fc (5 µg GO, 5 µg GDH, 0.25 µg EGDE), and (**D**) crosslinker EGDE (5 µg GO, 5 µg GDH, 30 µg LPEI-Fc). Amperometric measurements were performed at an applied potential of 0.35 V in 5mM NAD^+^ in PBS buffer pH 7.4, air-equilibrated electrolyte with the flow rate of 1.0 mL min^−1^.

The effects of varying the load of GO, the GDH enzyme, LPEI-Fc, and EGDE on the sensor performance were studied in a sequential manner for a few levels of each parameter to conduct a heuristic optimization as shown in Figure 4. The current signal was recorded after injecting 40 mM glucose in the FI system at a potential of 0.35 V. We chose this potential to achieve a sufficiently high current. Notice that the applied potential must be more positive than that of the redox polymer to enable the electron transport process. However, very high potentials should be avoided to prevent direct oxidation of NADH, electrode fouling, and secondary oxidation reactions. The optimized component loads for electrode modification are summarized in Table 2.

#### 3.4.1. Effect of GO Loading

The GO loading on the SPE surface was varied from 2.5 to 10 µg by drop-casting. Although some GO on the SPE can increase the electroactive surface area, an excessive amount could block the electrode and decrease the current response. As shown in Figure 4A, upon increasing the GO loading from 2.5 to 5 µg, the current response almost doubled. However, when the GO loading was further increased (>5 µg) the current response decreased. Therefore, we selected a GO load of 5 mg for further optimizations.

#### 3.4.2. Effect of GDH

Next, we optimized the GDH loading on the GO/SPE. While a higher loading of GDH will increase the number of reaction centers on the electrode, GDH is a non-conductive material. Thus, an excessive amount could decrease the current response. Indeed, this is observed in Figure 4B. By increasing GDH from 2 µg to 5 µg, the catalytic current increased, but when the GDH load exceeded 5 µg, the current started to decrease. Therefore, 5 µg of GDH was chosen for further experiments.

#### 3.4.3. Effect of Redox Polymer

An optimum LPEI-Fc amount was investigated by varying the polymer loading from 20 to 40 µg. The results are shown in Figure 4C. An increase in polymer loading from 20 µg to 30 µg led to an improvement in catalytic current. However, a higher loading seemed to hinder substrate diffusion across the polymer film. Thus, a loading of 30 µg LPEI-Fc was selected.

#### 3.4.4. Effect of EGDE Crosslinker

EGDE is a bi-functional crosslinker whose epoxy groups react with amino groups in the polymer backbone and increases the mechanical stability of the GDH–polymer film [55,56]. We varied the amount of EGDE introduced from 0.25 to 2 µg. The results, shown in Figure 4D, reveal that the crosslinker rapidly decreases the catalytic current. While the stability of the film was enhanced via crosslinking, its flexibility decreased, which may impact the rates of electron transfer and substrate diffusion. We compared the stability of the current signal obtained with 0.25 and 0.5 µg EGDE (Figure S2). Although the initial current response with 0.25 µg was higher, the electrode response with 0.5 µg was more stable after several measurements. Therefore, 0.5 µg was selected as the EGDE loading.

### 3.5. Effect of Flow Rate on Peak Current and Sample Throughput

**Figure 5:**
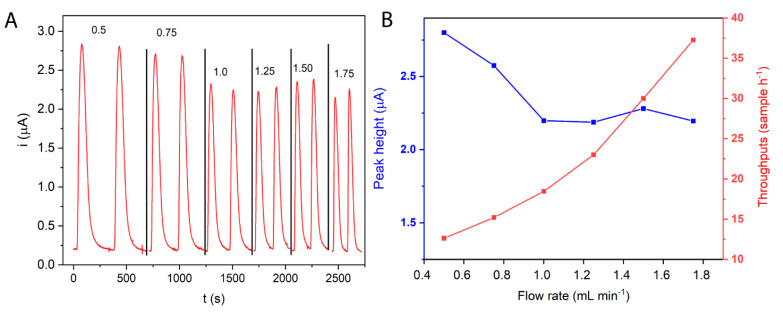
(**A**) Amperometric signal from glucose injection at varying flow rates (annotated numbers indicate the flow rate in mL min^−1^). (**B**) Effect of the operating flow rate on the current response peak heights and sample throughput. Measurements were conducted with the FIGDH/LPEI-Fc/GO/SPE sensor for a 40 mM glucose in PBS buffer pH 7.4.

The effect of the flow rate on the signal from glucose oxidation was investigated by injecting 40 mM glucose solution at various flow rates. As shown in Figure 5B, low flow rates provided a higher signal; however, they also reduced sample throughput. Therefore, we selected 1.0 mL min^−1^ to have sufficient sensitivity and reasonable sample throughput [47].

### 3.6. Calibration Graph and Analytical Features

**Figure 6:**
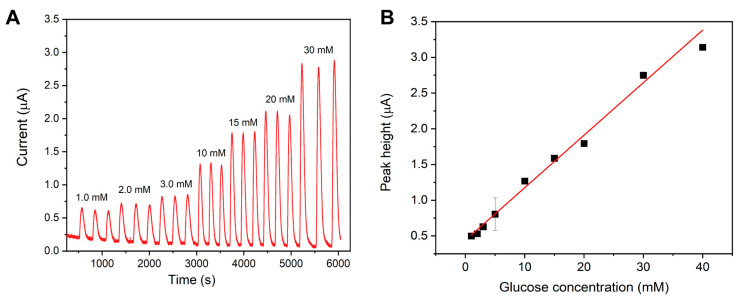
(**A**) Signals obtained from glucose at various concentrations (1.0–30 mM) measured with the optimized FI−GDH/LPEI-Fc/GO/SPE amperometric setup. (**B**) Linear calibration curve obtained between the peak height and glucose concentration. The operating conditions were as follows: applied voltage = 0.35 V; electrolyte = 5 mM NAD^+^ in PBS buffer pH 7.4; and air-equilibrated electrolyte; flow rate = 1.0 mL s^−1^.

The optimized FI-GDH/LPEI-Fc/GO/SPE sensor was used to analyze samples of varying glucose concentrations. The amperogram for standard glucose solutions between 1.0 and 80 mM is depicted in Figure 6A. We observe a sublinear dependence, which may be attributed to saturating reaction kinetics taking place on the electrode (e.g., hyperbolic Michaelis–Menten, see also [57]). Peak height values were obtained upon subtracting the baseline. A linear calibration curve (Figure 6B) was obtained for the concentration range explored as follows: y = 0.07355X + 0.4398 for X ∈ [1.0, 40.0] mM (R^2^ = 0.986), where y is baseline-subtracted peak height in µA and X is the glucose concentration in mM. The limit of detection (LOD) was estimated from 3S_b_/slope was 0.28 mM, where S_b_ is the standard deviation of blank measurements. Table 3 surveys some electrochemical glucose biosensors based on NAD-GDH in the previous literature. Importantly, the physiological range of glucose concentration is 2.8–22.2 mM [58], which is well covered by the proposed sensor. The quantification range of our sensor also compares well to that of commercial glucometers (0.6–33.3 mM).

### 3.7. Reproducibility, Repeatability, and Interference Studies

The reproducibility of the optimized design (Table 2) and repeatability of measurements were also examined by recording three replicate injections from five electrodes prepared independently on the same day with a 10 mM glucose solution. This resulted in a relative standard deviation (RSD) across all measurements of 10.7%. For a given electrode, the average repeatability for successive measurements was 5% RSD (Table S1).

The effect of potentially interfering substances on the sensor readout was investigated by adding a 10-fold excess (100 mM) of different sugars, including fructose, sucrose, xylose, and ribose, in the presence of 10 mM glucose as the analyte. The results, summarized in Table S2, show no significant interfering effect from these compounds (<5% deviation).

We also explored the potential impact of ascorbic acid on the measurements (Table S2). In this case, the interference was substantial. This is not surprising since ascorbic acid is capable of oxidation at a low applied potential [65]. This should not be a problem for the measurement of glucose in many beverages or, particularly, in plasma, where the concentration of glucose is more than two orders of magnitude higher than that of ascorbic acid [66,67]. However, it is a potential interferer that must be kept in mind. One workaround for samples with high ascorbate levels could be to use the electrode in potential measurement mode. In this case, instead of applying a voltage to the working electrode, the open circuit potential (OCP) could be measured, which would depend on the enzymatic reaction, and, therefore, the glucose concentration, but not on the non-enzymatic oxidation of ascorbic acid.

### 3.8. Application of the Proposed System to Real Samples Analysis

The proposed FI-GDH/LPEI-Fc/GO/SPE sensor was used to analyze the glucose concentration in a sports drink sample (Table 4). The sample was diluted 25-fold with 5 mM NAD^+^ in PBS buffer pH 7.4. The sample glucose concentration was quantified using the standard addition method [68]. The results obtained are in excellent agreement with the readout from the commercial glucometer, demonstrating that the sensor design can accurately determine the glucose concentration in a real sample.

## 4. Conclusions

We demonstrated the fabrication and application of a glucose biosensor based on a composite of graphene oxide and ferrocene-modified linear poly(ethylenimine) (LPEI-Fc) to improve the electron transfer from the glucose oxidation catalyzed by NAD-GDH. The sensor can be prepared in a facile manner by drop-casting on a screen-printed electrode. Thanks to the favorable electrostatic interaction between graphene oxide and LPEI-Fc, which possess opposite charges, the catalytic current extracted from NAD-GDH was significantly enhanced. The sensor was integrated into a simple flow injection system for amperometric sensing at a constant applied potential of 0.35 V. The sensor responded linearly to glucose concentration across a wide concentration range. The sensor exhibits good selectivity towards glucose and no significant interference from other carbohydrates. The sensor also provided good reproducibility and repeatability. The device was applied to analyze a sports drink, and the glucose measurement agreed well with the stated sugar content as well as an independent readout from a commercial glucometer. Given its performance, the electrode design might also be suitable as the bioanode for a biofuel cell-based self-powered sensor.

## Figures and Tables

**Table 1:** Comparison of the charge transfer resistance (R_ct_) measurements for different modified electrodes.

Electrode	R_ct_ (Ω)
Bare SPE	1745
GO/SPE	191
LPEI-Fc/GO/SPE	0.1
GDH/LPEI-Fc/GO/SPE	7.9

**Table 2:** Optimized component loads for electrode modification.

Component	Weight Loaded (µg)
GO	5
GDH	5
LPEI-Fc	30
EGDE	0.5

**Table 3:** Comparison of the proposed FI glucose sensing system’s performance with that of previously reported biosensors based on the NAD-GDH enzyme.

Electrode Modification	Detection Mode	E_app_ (V)	Linear Range	Detection Limit	Ref.
GDH/[Ru(bpy)_3_]^2+^-doped in silica sol–gel film/ITO	ECL/batch	1.3	25–2000 µM	0.5 µM	[59]
GDH/FePhenTPy/rGO/SPCE	Amp/batch	0.55	1.67–24 mM	0.67 mM	[4]
GDH/Medola blue/poly (ester sulfonic acid)	Amp/FI	0.10	0.15–20 mM	0.080 mM	[60]
GDH/Osphendione/CPE	Amp/batch	0.15	0.2–20 mM	-	[61]
GDH/Poly-HT/PAMAM/GCE	Amp/FI	0.30	0.005–1.0 mM	1.5 µM	[62]
PPF/GDH/PPF/Au	Amp/batch	0.60	2.5–26 mM	-	[63]
GDH/Os(bpy)_2_Cl_2_/DI/ITO	Amp/batch	0.00	0.1–30 mM	0.2 mM	[26]
GDH/poly(TB)/ERGO/GCE	BFC/batch	-	0.1–0.7 mM	-	[64]
GDH/LPEI-Fc/GO/SPE	Amp/FI	0.35	1.0–40 mM	0.28 mM	This work

ECL = electrochemiluminescence; Amp = amperometry; BFC = biofuel cell; ITO = indium tin oxide; GCE = glassy carbon electrode; Au = gold electrode; CPE = carbon paste electrode; [Ru(bpy)_3_]^2+^ = tris(2,2′-bipyridyl)ruthenium(II) FePhenTyr = 5-[2,5-di (thiophen-2-yl)-1H-pyrrol-1-yl]-1,10-phenanthroline iron(III) chloride; PAMAM = Poly-amidoamine; Osphendione = Os(4,4′-dimethyl, 2,2′-bipyridine)_2_(1,10-phenanthroline-5,6-dione); Poly-HT = Poly-Hematoxylin; PPF = plasma-polymerized thin film, rGO = reduced graphene oxide; ERGO = electrochemically reduced graphene oxide; DI = Diaphorase; Os(bpy)_2_Cl_2_ = bis(2,2-bipyridyl)dichloro osmium(II); Poly(TB) = poly-Toluidine blue.

**Table 4:** Application of the proposed system to a real sample.

Sample	FI System (g dL^−1^)	Glucometer (g dL^−1^)	Label (g dL^−1^)
Sports drink	1.69 ± 0.03	1.68 ± 0.47	1.7

## Data Availability

The data that support the findings of this study are available from the corresponding authors upon reasonable request.

## Supplementary Materials

The following supporting information can be downloaded at: https://www.mdpi.com/article/10.3390/bios14040161/s1, Figure S1: FT-IR spectra of (a) graphite powder and (b) GO.; Figure S2: Comparison of the electrode current responses to repeated measurements of 40 mM glucose.; Table S1: Amperometric signal values of 10 mM glucose obtained from five independent biosensors for reproducibility and repeatability studies.; Table S2: Effect of potentially interfering substances on the FI-GDH/LPEI-Fc/GO/SPE amperometric response to 10 mM glucose.

## Abbreviations

EGDE	ethylene glycol diglycidyl ether
FI	flow injection
GDH	glucose dehydrogenase
GOx	glucose oxidase
GO	graphene oxide
NAD^+^	nicotinamide adenine dinucleotide (oxidized form)
NADH	nicotinamide adenine dinucleotide (reduced form)
FAD	flavin adenine dinucleotide
PQQ	pyrroloquinoline quinone
LPEI-Fc	ferrocene-modified linear poly(ethylenimine)
SPE	screen-printed electrode
PBS	phosphate-buffered saline
