# Supplementary material for: Enhancing Glucose Biosensing with Graphene Oxide and Ferrocene-Modified Linear Poly(ethylenimine)
Source: Biosensors (Basel). 2024 Mar 28;14(4):161. doi: 10.3390/bios14040161 (PMC11048651; doi:10.3390/bios14040161)
Supplement: Supplementary file 1 [file biosensors-14-00161-s001.zip › biosensors-2849744-supplementary.pdf]

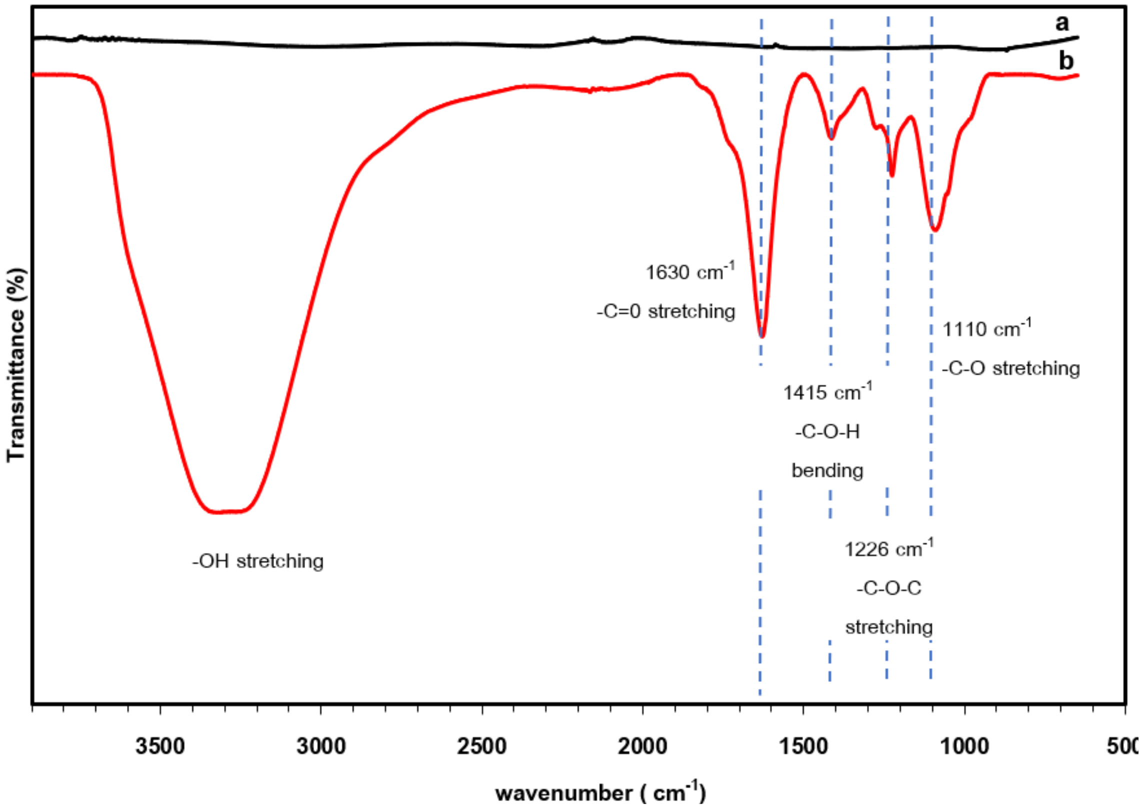

Figure S1. FT-IR spectra of a) graphite powder and b) GO.

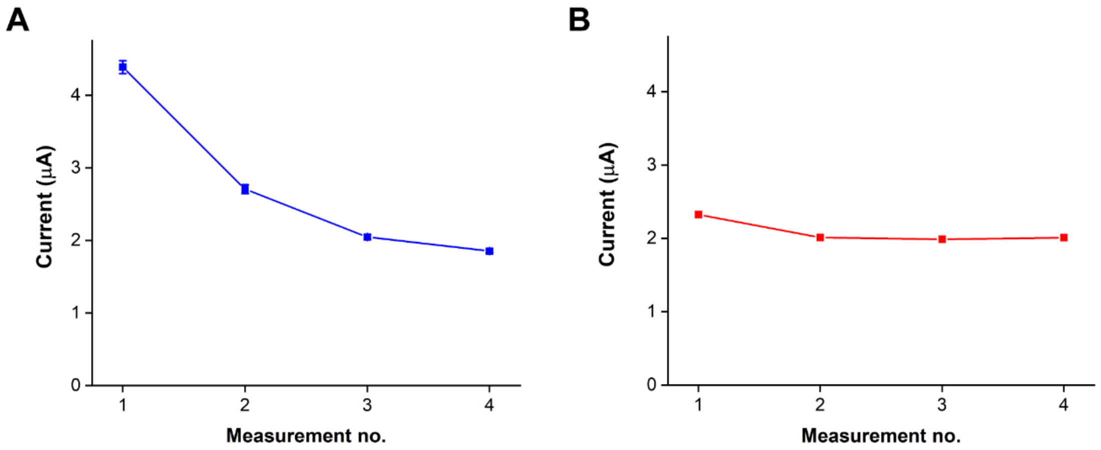

Figure S2. Comparison of the electrodes current responses to repeated measurements of 40 mM glucose. A) 0.25 µg EDGE, B) 0.50 µg EDGE. (5 µg GO, 5 µg GDH, 30 µg LPEI-Fc). Measurements were performed at an applied potential of 0.35 V in 5mM NAD^+ in PBS buffer pH 7.4, air-equilibrated electrolyte with the flow rate of 1.0 mL/min.

Table S1. Amperometric signal values of 10 mM glucose obtained from five independent biosensors for reproducibility and repeatability studies.

Electrode no.	Current (μA)	*%RSD* (from the same sensor)
1	1.378	8.37
	1.616	
	1.576	
2	1.260	1.99
	1.217	
	1.217	
3	1.636	5.93
	1.522	
	1.713	
4	1.588	6.10
	1.407	
	1.525	
5	1.408	4.16
	1.345	
	1.462	
Average	**1.458**	**5.31**
Standard deviation	***0.156***	
%RSD	***10.70***	

Table S2. Effect of potentially interfering substances on the FI-GDH/LPEI-Fc/GO/SPE amperometric response to 10 mM glucose.

Substance	%deviation
100 mM Fructose	0.11
100 mM Sucrose	0.91
100 mM Xylose	1.21
100 mM Ribose	2.85
100 mM Caffeine	1.14
1.0 mM Ascorbic acid	15.79
